# A Family Relationships Class Project: Creating Podcast Episodes From Older Adult Interviews

**DOI:** 10.1093/geroni/igaf122.1588

**Published:** 2025-12-31

**Authors:** Melinda Heinz

**Affiliations:** University of Northern Iowa, Cedar Falls, Iowa, United States

## Abstract

Students enrolled in a Family Relationships course created a podcast episode based on three interviews with older adults. After asking students to complete self-reflections on interviewing and technology strengths, student pairs were created, matching strong interview skills with savvy technology abilities. Together, students generated questions about what they might ask their older adult, focusing on family relationships. Students met their older adult interviewees on Zoom and recorded the meetings. After the first interview, each group discussed what the theme of their podcast episode might be and proceeded to ask follow-up questions at their next two meetings with the older adult. Student pairs were also required to act as podcast hosts by recording their own dialogue with each other, introducing content, navigating to interview segments with the older adult, and expanding on topics mentioned by the older adult and relating it to course content. At the end of the semester, each student pair created a singular podcast episode that was shared and discussed in class. Overall, students reported that they enjoyed the creativity and agency with creating their own podcast episode, but at times faced barriers with technology. Students also reported that they enjoyed learning how family relationships evolved and changed over time (e.g., role reversal with caring for a parent) directly from the older adult. Instructors may want to consider partnering with another class who has more advanced video editing skills or bringing a guest in to teach basic audio editing techniques.

